# Impaired cognition predicts the risk of hospitalization and death in cirrhosis

**DOI:** 10.1002/acn3.50924

**Published:** 2019-10-20

**Authors:** Minjee Kim, Eric M. Liotta, Phyllis C. Zee, Daniel R. Ganger, Daniela P. Ladner, Ameeta Karmarkar, John D. Peipert, Farzaneh A. Sorond, Shyam Prabhakaran, Kathryn J. Reid, Andrew M. Naidech, Matthew B. Maas

**Affiliations:** ^1^ Division of Stroke and Neurocritical Care Department of Neurology Northwestern University Chicago Illinois; ^2^ Center for Circadian and Sleep Medicine Department of Neurology Northwestern University Chicago Illinois; ^3^ Northwestern University Transplant Outcomes Research Collaborative (NUTORC) Comprehensive Transplant Center (CTC) Northwestern University Chicago Illinois; ^4^ Division of Organ Transplantation Department of Surgery Northwestern University Chicago Illinois; ^5^ Division of Gastroenterology and Hepatology Department of Medicine Northwestern University Chicago Illinois; ^6^ Department of Medical Social Sciences Northwestern University Chicago Illinois; ^7^ Department of Neurology University of Chicago Chicago Illinois

## Abstract

**Objective:**

Cognitive impairment, detected in up to 80% of patients with liver cirrhosis, is associated with negative health outcomes but is underdiagnosed in the clinical setting due to the lack of practical testing method. This single‐center prospective observational study aimed to test the feasibility and prognostic utility of in‐clinic cognitive assessment of patients with liver cirrhosis using the NIH Toolbox cognition battery (NIHTB).

**Methods:**

Patients recruited from a hepatology/transplant clinic underwent cognitive assessments using West‐Haven Grade (WHG) and NIHTB between November 2016 and August 2018 and were prospectively followed until December 2018. The primary outcome was a composite end point of hospitalization related to overt hepatic encephalopathy (OHE) and all‐cause mortality during follow‐up, evaluated by a Cox proportional hazards regression model that adjusted for *a priori* covariates (age and MELD‐Na).

**Results:**

Among 127 patients (median age 60 years, 48 [38%] women) assessed, cognitive performance was significantly impaired in 82 [78%] patients with WHG 0 and 22 [100%] patients with WHG 1 and 2. Over a median of 347 days follow‐up, 18 OHE and 8 deaths were observed. Lower cognitive performance was associated with an increased risk of OHE/death adjusting for age and MELD‐Na. Subclinical cognitive impairment detected by NIH Toolbox in WHG 0 patients was significantly associated with greater mortality. Median time to complete the two prognostically informative NIH Toolbox tests was 9.4 min.

**Interpretation:**

NIH Toolbox may enable a rapid cognitive screening in the outpatient setting and identify patients at high risk for death and hospitalization for severe encephalopathy.

## Introduction

Hepatic encephalopathy (HE) is a prevalent and disabling complication of liver cirrhosis.^1^ The clinical form of HE, overt HE (OHE), is the most common potentially preventable cause of hospitalization among patients with cirrhosis, with an estimated $1.3 billion annual healthcare cost in the United States and 30‐day mortality ranging from 8% to 38%.^2^, ^3^ HE is now seen as a continuum from subclinical cognitive impairment, also known as covert or minimal HE, to the clinically apparent OHE where arousal is affected to various degrees.^4^ Covert HE is characterized by subtle cognitive impairment that can only be detected through specialized psychometric testing and has been detected in up to 80% of tested patients.^5^, ^6^ Covert HE is associated with the risk of OHE and death in patients with cirrhosis, despite controlling for the Model for End‐stage Liver Disease (MELD) score.^7^, ^8^, ^9^

Given this prognostic importance, North American and European liver disease societies have recommended screening for cognitive impairment in the outpatient setting.^10^ However, only 10% of clinicians routinely screen for cognitive impairment due to the lack of time, personnel, and consensus on testing method.^11^, ^12^, ^13^ Various neuropsychological and neurophysiological methods used in research settings to screen cognitive impairment failed to gain broad clinical use for two reasons: the cost of equipment or trained personnel combined with long test duration that makes performance impractical, and the lack of clear consensus on the threshold to define an abnormal result relative to the general population.^7^, ^8^, ^14^, ^15^, ^16^ The need for a practical, standardized, and cost‐effective method that is readily available for busy clinicians to screen for cognitive impairment is widely recognized in the field.^16^

The NIH Toolbox (NIHTB) is a brief, royalty‐free, standardized, and validated method to measure various domains of neurological function on a tablet, which has rapidly gained popularity in a broad spectrum of neuroscience research and has been the method of choice for NIH funded studies.^17^ The NIHTB cognition battery consists of seven individually validated tests, each of which can be completed in shorter time with equal or better precision compared to paper‐and‐pencil alternatives due to computerized adaptive testing.^18^

While the NIHTB has been validated against individual components of the psychometric hepatic encephalopathy score (PHES), a four‐test paper‐and‐pencil battery that has been considered the gold standard method to diagnose covert HE,^14^ the utility of NIHTB in patients with cirrhosis is unknown. In this single‐center, prospective cohort study of adults with cirrhosis who are evaluated for liver transplant, we hypothesized that (1) a routine cognitive assessment using the NIHTB is feasible in the busy clinical setting, and (2) the NIHTB can identify patients who are at risk of OHE‐related hospitalization and death, independent of age and liver disease severity.

## Methods

### Study population

The Hepatic Encephalopathy Registry of patients with liver Cirrhosis Undergoing Liver transplant Evaluation Steps (HERCULES) is an ongoing prospective study of adult patients with cirrhosis who were enrolled between November 2016 and August 2018 by random sampling from pre‐transplant liver clinic at Northwestern Memorial Hospital in Chicago, IL, USA. All patients had a proven diagnosis of cirrhosis based on radiographic, biochemical, or histological findings, with significant clinical manifestations to warrant evaluation for liver transplant, as determined by the multidisciplinary transplant team. English‐speaking men and women 18 years of age and older who had been free of alcohol or illicit drugs for 6 months were eligible. Patients with severe extrahepatic organ dysfunction (e.g., end‐stage renal disease requiring maintenance renal replacement therapy or glomerular filtration rate < 30 mL/min/1.73 m^2^), a history of primary neurological disorders such as stroke or traumatic brain injury, and terminal illness of non‐hepatic origin were excluded. The study was approved by the Northwestern University Institutional Review Board. Written informed consent or waivers of informed consent were obtained from all participants according to the approved protocol.

### Measurements of baseline characteristics

Patients underwent baseline cognitive assessment using a pre‐defined set of the NIHTB Cognition Battery: Pattern Comparison Processing Speed test (PCPS), Flanker Inhibitory Control and Attention test (FICA), List Sort Working Memory test (LSWM), and Dimensional Change Card Sort test (DCCS). The FICA and DCCS are tests of executive function, while PCPS and LSWM test processing speed and working memory, respectively. These four tests were selected to examine domains of cognition that are most frequently affected in covert hepatic encephalopathy, similar to the PHES.^14^ (For details on each test, see Data S1).

Each of these NIHTB tests has been shown to be valid and reliable.^17^ For each test, raw scores are automatically transformed to a demographically‐corrected (age, education, gender, and race) T‐score normed to a U.S. general population mean of 50 and a standard deviation (SD) of 10.^18^ Clinical impairment in a specific test was defined as greater than one SD below the normed mean (i.e., demographically‐corrected T‐score < 40), based on the Institute of Medicine practice guidelines on the interpretation of standardized cognitive test performances.^19^, ^20^ NIHTB tests were administered on iPad tablet in the clinic by one of two study team members who underwent online training offered at the NIHTB website (http://www.healthmeasures.net/explore-measurement-systems/nih-toolbox).

Structured interviews were conducted to determine present encephalopathy by West Haven Grade (WHG) as follows: 0 = asymptomatic, 1 = any alteration in mentation, 2 = lethargy, disorientation, or presence of asterixis, 3 = arousable with difficulty, and 4 = unresponsive to deep pain. WHG grader was blinded to NIHTB results and paper‐and‐pencil tests. Same‐day laboratory values were obtained to calculate the Model for End‐Stage Liver Disease—Sodium (MELD‐Na) scores, per standard liver transplant evaluation protocol. Patients with active decompensation requiring hospitalization were excluded from participation until they achieved a full recovery. All patients had negative blood alcohol level and urine toxicology. Medical records were reviewed to confirm history and to obtain additional details regarding medications, comorbidity, and the etiology, severity, and complications of cirrhosis.

While the NIHTB tests used in this study have been validated against commonly used psychometric tests, including the Stroop test and all components of the PHES,^17^ we administered a set of paper‐and‐pencil psychometric tests in a subset of patients to confirm the validity of the NIHTB tests, including mini‐mental status examination (MMSE), number connection tests A/B (NCT‐A/B), and digit symbol test (DST).^17^, ^21^, ^22^, ^23^ NCT and DST are widely available tests that are also included in the PHES.^14^ Individuals administering and scoring paper‐and‐pencil psychometric tests were blinded to the WHG or NIHTB findings.

### Outcome measures

Patients were followed up prospectively at Northwestern Memorial Hospital. All patients were regularly seen in the clinic at 1 – 6‐month intervals per standard of care and were tracked via electronic medical system for any liver‐related complications, hospitalizations, transplant, and death. Time from initial screening to the first OHE‐related hospitalization and death were recorded in days. Primary outcome was a prespecified composite end point of OHE‐related hospitalization and all‐cause mortality. Prospective patient data until December 1, 2018 were retrospectively reviewed and analyzed.

### Statistical analysis

Continuous variables were described as mean ± SD for normally distributed variables and as median (interquartile range) for non‐normally distributed variables. Categorical variables were described with frequencies and proportions. Categorical variables were compared using chi‐squared or Fisher’s exact tests, whereas continuous variables were compared using *t*‐tests (for normally distributed variables) or Mann–Whitney U‐tests (for non‐normally distributed variables). A 2‐sided *P < *0.05 was considered significant. Correlations were assessed using Spearman’s correlation coefficient.

Linear regression was used to identify factors independently associated with cognitive function. The pre‐specified prognostic variables included in linear regression model were: age, gender, NIHTB T‐score (individual tests and average of all tests), history of OHE, etiology of cirrhosis, transjugular intrahepatic portosystemic shunt (TIPS), complete blood count, and MELD‐Na score. We used a backward variable selection algorithm based on likelihood ratio to identify a parsimonious set of prognostic variables. For time‐to‐event analysis, age and MELD‐Na were selected *a priori* as covariates for Cox proportional hazards regression model for a composite end point of death or OHE‐related hospitalization, based on both biological mechanism and evidence from previously published studies.^7^, ^8^ For patients who experienced both death and OHE‐related hospitalization during follow‐up, the earliest event was used for time‐to‐event analysis. Patients who received liver transplant were censored at the time of transplant. All analyses were performed in R version 3.5.1 (R Foundation for Statistical Computing, Vienna, Austria). Time‐to‐event analyses were conducted with the “survival” and “survminer” packages.^24^, ^25^, ^26^

## Results

### Demographic

One hundred and twenty‐seven patients were followed for a median of 347 (interquartile range [IQR] 111‐536) days. One hundred patients completed the four‐test NIHTB, and 27 patients partially completed the NIHTB tests (3/4 tests in 23 patients and 2/4 tests in four). The most common reason for missing data was insufficient time to complete the study procedures. Fifty patients completed additional paper‐and‐pencil tests.

Patient characteristics are summarized in Table 1. Among 127 patients who were included in the analysis, the median age was 60 (54‐65) years, 48 (38%) were women, and the median MELD‐Na was 13 (9‐17.5). 75 (59%) patients were on treatment for OHE.

**Table 1:** Baseline characteristics of the patients (*n* = 127).

Variables	
Age, years	60 [54–65]
Female sex, *N* (%)	48 (38)
Education, years	14.0 ± 2.6
Race, white/black/Asian/other, *N *(%)	102 (80)/8 (6)/8 (6)/17 (6)/8 (6)
Hispanic, *N* (%)	17 (13)
BMI	30.7 ± 6.8
NIHTB demographic‐adjusted T‐score	
FICA	36.7 ± 7.6
DCCS	44.5 ± 10.4
LSWM	43.2 ± 10.5
PCPS	38.1 ± 13.6
Average	40.5 ± 8.6
WHG 0/1/2, *N *(%)	105 (83)/14 (11)/8 (6)
MELD‐Na	13 [9–17.5]
Leukocyte count, K/L	5.1 ± 2.1
Hemoglobin, g/dL	12.3 ± 3.4
Platelets, K/L	108.1 ± 66.0
Sodium, mEq/L	137.3 ± 3.9
Creatinine, mg/dL	1.0 ± 0.5
INR	1.3 ± 0.3
Bilirubin, total, mg/dL	2.5 ± 1.9
Albumin, g/dL	3.4 ± 0.6
TIPS, *N* (%)	21 (17)
Etiology of cirrhosis, alcohol/viral/NASH/other, *N *(%)	49 (39)/36 (28)/27 (21)/15 (12)
Hepatocellular carcinoma, *N* (%)	27 (21)
Current treatment for HE, *N* (%)	75 (59)
Lactulose, *N* (%)	65 (51)
Rifaximin, *N* (%)	62 (49)
Both, *N* (%)	52 (41)
History of cirrhosis complications	
OHE, *N* (%)	75 (59)
Hepatorenal syndrome, *N* (%)	16 (13)
Hepatopulmonary syndrome, *N* (%)	8 (6)
Peritonitis, *N* (%)	12 (9)
Hyponatremia, *N* (%)	76 (60)
Ascites, *N* (%)	87 (69)
Variceal bleed, *N* (%)	91 (72)
Comorbidities	
Diabetes, *N* (%)	38 (36)
Hypertension, *N* (%)	78 (54)
Hyperlipidemia, *N* (%)	29 (23)
Sleep apnea, *N* (%)	23 (18)

Data are presented as mean ± SD for normally distributed variables median [interquartile range] for non‐normal variables unless otherwise noted.

BMI, Body mass index; MELD, model for end‐stage liver disease; NASH, non‐alcoholic steatohepatitis; NIHTB, National Institute of Health Toolbox for the Assessment of Neurological Behavior and Functions; DCCS, Dimensional Change Card Sort; FICA, Flanker Inhibitory Control and Attention; LSWM, List Sorting Working Memory; PCPS, Pattern Comparison Processing Speed; OHE, overt hepatic encephalopathy; TIPS, transjugular intrahepatic portosystemic shunt; WHG, West‐Haven Criteria Grade.

### Baseline assessment

**Figure 1:**
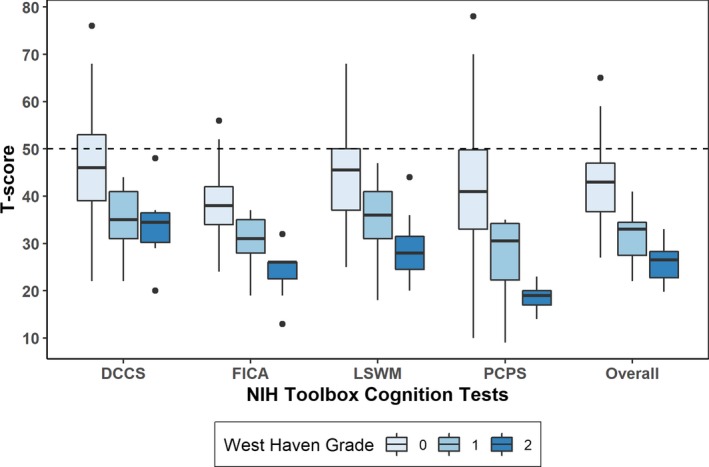
NIH Toolbox Cognition Test Results by West Haven Grade. Boxplots of T‐scores are shown for each test within the NIH Toolbox Cognitive Battery along with the mean overall score, divided by patients’ West Haven Grade. Dashed line represents demographic‐matched population norm. DCCS‐Dimensional Change Card Sort, FICA‐Flanker Inhibitory Control and Attention, LSWM‐List Sorting Working Memory, and PCPS‐Pattern Comparison Processing Speed.

By interview, 105 (83%) were graded as WHG 0 (asymptomatic), 14 (11%) as WHG 1 (any alteration in mentation), and 8 (6%) as WHG 2 (lethargy, disorientation, or asterixis). All patients graded as WHG 1/2 were on treatment for OHE. The median assessment time for the four‐test NIHTB battery was 15.6 (10.3‐17.0) minutes. The median assessment time for individual NIHTB tests ranged from 1.7 (1.6–1.7) minutes for PCPS to 5.9 (5.6‐6.8) minutes for LSWM. The mean NIHTB T‐score was 40.5 ± 8.6 (one‐sample *t*‐test *P* < 0.001 vs. the general population adjusted for age, education, gender, race/ethnicity with mean of 50 ± 10). NIHTB T‐scores are summarized in the Figure 1 for each test, stratified by WHG. Patients with no obvious impairment by interview (WHG 0) scored significantly worse than the demographically matched general population in all four NIHTB tests (*P* < 0.001), and cognitive scores declined according to WHG (all *P* < 0.001). Cognitive impairment (NIHTB T‐score < 40 in one or more NIHTB tests) was identified in 82 (78%) WHG 0 patients and 22 (100%) WHG 1/2 patients. Patients with a history of OHE performed worse than patients without a history of OHE (average T‐score 37.7 ± 7.92 vs. 44.5 ± 7.94, *P* < 0.001). Individual T‐scores of the four NIHTB tests were correlated with each other (Spearman’s rho 0.44‐0.61, all *P* < 0.001).

In univariate analyses, average NIHTB T‐score was significantly associated with history of OHE (37.8 vs. 44.5, *P* < 0.001), albumin (rho 0.33, *P* < 0.001), international normalized ratio (INR, rho −0.33, *P* < 0.001), and MELD‐Na (rho −0.26, *P* = 0.004), but was not significantly associated with age, gender, education, leukocyte count, hemoglobin, platelets, sodium, creatinine, bilirubin, etiology of cirrhosis*,* or TIPS. Only history of OHE (B −6.1, 95% confidence interval [−9.1,−3.1], *P* < 0.001) and INR (B −11.8 [−17.9,−5.6], *P* < 0.001) was significantly associated with average NIHTB T‐score in multivariable modeling.

Concurrent NIHTB and paper‐and‐pencil test results were available in 50 patients. The NIHTB T‐scores were correlated with NCT‐A/B and DST (all *P* < 0.001 versus average T‐Score; see Table 2). In a sensitivity analysis, the subset of patients who completed baseline paper‐and‐pencil tests was not different from the rest of cohort regarding age, gender, MELD‐Na, education, and NIHTB T‐scores.

**Table 2:** Correlation between Paper‐and‐pencil Tests and the NIH Toolbox (*n* = 50).

Paper‐and‐pencil tests		Correlation with the NIH Toolbox T‐scores, Spearman’s rho (*P* value)				
Test	Results	FICA	DCCS	LSWM	PCPS	Average
Mini mental State Examination	29 [28–30]	0.18 (*P* = 0.3)	0.46 (*P* = 0.002)	0.60 (*P* < 0.001)	0.22 (*P* = 0.2)	0.46 (*P* = 0.002)
Number connection test A, seconds	38.1 [30.0–49.7]	−0.41 (*P* = 0.002)	−0.54 (*P* < 0.001)	−0.40 (*P* = 0.005)	−0.59 (*P* < 0.001)	−0.62 (*P* < 0.001)
Number connection test B, seconds	80.0 [60.8–154.2]	−0.46 (*P* < 0.001)	−0.38 (*P* = 0.02)	−0.40 (*P* = 0.005)	−0.44 (*P* = 0.002)	−0.56 (*P* < 0.001)
Digit symbol test, points	35 [28–44]	0.60 (*P* < 0.001)	0.65 (*P* < 0.001)	0.60 (*P* < 0.001)	0.59 (*P* < 0.001)	0.75 (*P* < 0.001)

Data are presented as median [interquartile range] for non‐normally distributed variables.

DCCS, Dimensional Change Card Sort; FICA, Flanker Inhibitory Control and Attention; LSWM, List Sorting Working Memory; PCPS, Pattern Comparison Processing Speed.

### Time to Death or OHE‐Hospitalization (Primary outcome)

**Figure 2:**
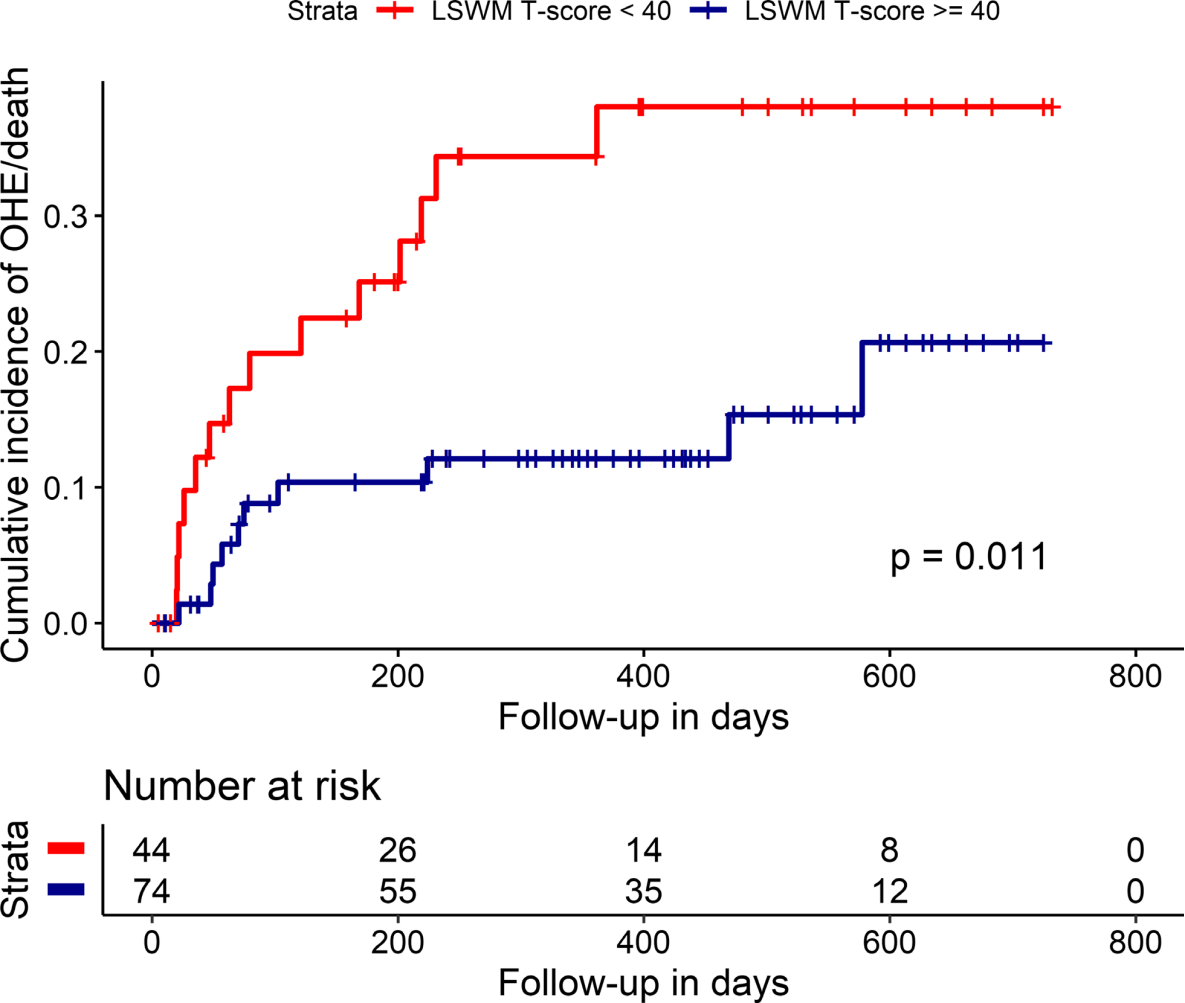
Time from cognitive assessment to overt hepatic encephalopathy (OHE)‐related hospitalization or death. Time from cognitive screening to OHE‐related hospitalization or death showed a significant difference between patients who scored one standard deviation below the demographic‐matched norm (T‐score < 40) in the NIH Toolbox List Sort Working Memory test and those who scored equal to or greater than 40. The Kaplan–Meier curve for estimated cumulative incidence of the primary end point in the Y axis and days from initial cognitive screening in the X axis. The red lines depict patients who scored below 40 at study entry, and blue lines denote those who scored equal to or greater than 40, with each vertical line signifying censoring. Log‐rank statistics were used to compare groups. LSWM‐List Sorting Working Memory, OHE‐Overt Hepatic Encephalopathy.

During the follow‐up period, prespecified primary end point events were observed in 26 (20.5%) unique patients, including 8 (6.3%) deaths and 18 (14.2%) OHE‐hospitalizations. Three patients who were hospitalized for OHE subsequently died (total 11 (8.7%) deaths). The median time until the primary end point from the time of cognitive assessment was 347 (111–536) days. Thirty‐six patients were censored at the time of liver transplant without experiencing the primary outcome event (median time until transplant = 135 [40–247] days). Univariate analysis identified that two NIHTB T‐scores (LSWM [*P* = 0.006] and FICA [*P* = 0.002]), MELD‐Na (*P* = 0.002), INR (*P* < 0.0001), bilirubin (*P* = 0.04), and albumin (*P* < 0.0001) were significantly associated with the time to death or OHE‐hospitalization. In a multivariable event‐free survival model, NIHTB LSWM T‐score (hazard ratio (HR) 0.93 [0.89, 0.97], *P* = 0.002) and FICA T‐score (HR 0.94 [0.89, 0.98], *P* = 0.009) were significantly associated with death or OHE‐hospitalization, adjusting for *a priori* covariates including age and MELD‐Na (see Fig. 2). In exploratory models adjusting for a history of OHE in addition to age and MELD‐Na, both NIHTB LSWM T‐score (HR 0.93 [0.89–0.98], *P* = 0.003) and FICA T‐score (HR 0.93 [0.88–0.99], *P* = 0.01) remained significantly associated with death or OHE hospitalization.

In multivariable analyses of individual components of the composite primary outcome, two NIHTB T‐scores (LSWM: HR 0.91 [0.85, 0.99], *P* = 0.02; FICA: HR 0.92 [0.85, 0.98], *P* = 0.02) were significantly associated with the time to death, and LSWM T‐score was significantly associated with the time to OHE‐hospitalization (HR 0.94 [0.89, 0.99], *P* = 0.02), adjusting for *a priori* covariates including age and MELD‐Na. Additional adjustment of the multivariable model including history of OHE as a covariate did not meaningfully change the results.

### Subclinical Cognitive Impairment and Time to Death or OHE‐Hospitalization

In patients with no obvious cognitive impairment by interview (WHG 0, *n* = 105), 13 (12.4%) OHE‐hospitalizations and 7 (6.7%) deaths were observed in 20 unique patients during the follow‐up period. Two patients died after OHE‐hospitalizations (total death = 9/105 (8.6%)). Among those WHG 0 patients, two NIHTB T‐scores were associated with the composite primary outcome of death or OHE‐hospitalizations in univariate analysis (LSWM [*P* = 0.004] and FICA [*P* < 0.001]) and multivariable models (LSWM: HR 0.92 [0.86, 0.97], *P* = 0.003; FICA: HR 0.89 [0.83, 0.95], *P* < 0.001) adjusting for *a priori* covariates including age and MELD‐Na. In exploratory models including history of OHE as a covariate in addition to age and MELD‐Na, LSWM and FICA T‐scores remained significantly associated with death/OHE‐hospitalizations and of similar effect size in WHG 0 patients.

In multivariable analyses of individual components of the primary outcome, two NIHTB T‐scores were associated with death (LSWM: HR 0.86 [0.77, 0.96], *P* = 0.007; FICA: HR 0.80 [0.71, 0.91], *P* < 0.001) in WHG 0 patients, adjusting for age and MELD‐Na; this is equivalent to HR 4.4 [1.5, 13.1] and HR 9.2 [2.7, 31.5] for death per standard deviation decrease in LSWM T‐score and FICA T‐score, respectively. In exploratory models adjusting for history of OHE in addition to age and MELD‐Na, the association with death did not change meaningfully (LSWM: HR 0.86 [0.78–0.96], *P* = 0.009; FICA: 0.79 [0.70–0.90], *P* < 0.001). NIHTB T‐scores were not significantly associated with OHE‐hospitalization in WHG 0 patients (LSWM: HR 0.94 [0.88, 1.01]; FICA: HR 0.92 [0.85, 1.01]), after adjusting for age and MELD‐Na.

## Discussion

In this single‐center prospective cohort study, rapid cognitive assessment using the four‐test NIHTB battery was feasible in a busy transplant clinic, with 97% completion rate for three or more tests. Additionally, lower NIHTB scores were associated with the risk of OHE/death independent of age or MELD‐Na, based on two NIHTB tests that can be completed in under 10 min. As expected in an ambulatory setting, a majority (83%) of patients appeared unimpaired by WHG, whereas NIHTB tests detected subclinical cognitive impairment in 78% of WHG 0 patients, defined as one standard deviation lower than the demographic‐matched population norm. In this group, subclinical cognitive impairment was associated with greater mortality. This association suggests that covert HE is a potential mortality risk factor in liver transplant candidates. Lastly, NIHTB tests were correlated with popular paper‐and‐pencil tests such as NCT and DST, constituents of the PHES, providing additional verification of their validity in this population beyond the validation studies already performed.

While the importance of subtle cognitive impairment, termed minimal or covert HE, has been widely recognized, most practitioners find it impractical to screen patients for cognitive impairment in the clinical setting due to the current lack of a rapid, standardized, and cost‐effective diagnostic method.^13^ The NIHTB has gained wide popularity in neuroscience research as a rapid, royalty‐free, and rigorously validated method. Major advantages of the NIHTB include a short test duration, low cost, and granular assessment that is automatically transformed to demographic‐adjusted T‐scores. Moreover the NIHTB is highly customizable. Among the four NIHTB tests used in this study, the tests for working memory (LSWM) and executive function (FICA) showed the strongest association with death or OHE. These two tests together can be administered in under 10 min by any clinic staff who completes the one‐hour online training, which increases the feasibility of routine cognitive screening in the clinic. (http://www.healthmeasures.net/NIH_Toolbox_iPad_e-learning).

Several previous reports have suggested that cognitive impairment predicts future development of OHE and death. Patidar et al. demonstrated that subclinical cognitive impairment, based on NCT‐A/B, DST, and block design, was associated with new onset OHE and death/liver transplant in cirrhotic patients without previous OHE.^7^ Hartmann et al. demonstrated increased risk of OHE, but not mortality, among patients with subclinical HE measured by NCT‐A, DST, and electroencephalogram.^15^ Ampuero et al. showed increased mortality in patients with untreated minimal HE, measured by critical flicker frequency.^8^ In line with these previous reports, we found increased mortality among patients with subclinical cognitive impairment detected by NIHTB. Additionally, the NIHTB provides a continuous and normalized score adjusted for patient’s demographic background, which is not only more informative than a binary result but also reflects the current understanding of HE as a continuum of brain dysfunction. In contrast to previous studies that excluded patients with prior OHE or concurrent use of rifaximin or ammonia‐lowering agents, 50% of our patients with subclinical cognitive impairment were on treatment for prior OHE, which reflects the pre‐transplant population with more advanced cirrhosis. In this study, cognitive impairment detected by the NIHTB was a significant predictor of the primary outcome even after adjusting for a history of OHE, suggesting that individual neurological vulnerability captured by contemporary cognitive function is informative in addition to a history of OHE. Therefore, this study adds to the existing literature that subclinical cognitive impairment in liver transplant candidates, either undiagnosed or partially treated, presents in a continuum, and a greater degree of cognitive impairment is associated with increased mortality.

This study has several limitations. First, this is a single center observational study. A process feasible in our system may encounter impediments elsewhere. Second, we did not validate the NIHTB against popular batteries such as the complete PHES battery or EncephalApp Stroop test,^14^, ^27^ as this pragmatic study focused on feasibility testing of the NIHTB in our institution where no previous testing tools had achieved a clinical application. However, the NIHTB tests were correlated with several components of the PHES in a subset of the study cohort. Both EncephalApp and NIHTB can rapidly assess psychomotor speed and cognitive flexibility.^27^ We chose the NIHTB battery in this study because it provides continuous, non‐binary scores that are easy to understand for clinicians and it is less susceptible to physical confounders such as tremor and poor motor control. Third, the electronic health records used in this study would not have captured hospitalizations outside of this health system. However, patients followed in this transplant program are usually transferred to this hospital for liver‐related complications, and missing data on hospitalizations that occur outside this health system are unlikely to be associated with patients’ baseline cognitive function. Fourth, this study included patients who routinely take psychoactive medications such as antidepressants and sleep aids, which are reported in up to half of patients with cirrhosis.^28^ Although psychoactive medications may influence test performance, it is unrealistic to deny patients such medications in practice for the purpose of accurately assessing cognitive performance. Fifth, our results may not be generalizable to all patients with cirrhosis. Over a half of the cohort had a history of OHE and 17% had persistent low‐grade OHE, reflecting the pre‐transplant clinic setting where patients with more advanced cirrhosis are referred for evaluation. Sixth, non‐English‐speaking patients were excluded, because non‐English versions of the NIHTB were not yet available in the beginning of the study period. Further research including non‐English‐speaking patients using the NIHTB is necessary to extend these findings into other languages.

In conclusion, these data support the validity of NIHTB for cognitive assessment of ambulatory patients with advanced liver disease, its feasibility for integration into routine outpatient care, and its utility for identifying patients at risk of OHE and death. Using this sensitive, quantitative cognition assessment instrument, we further identified a novel association between subclinical cognitive impairment and mortality, emphasizing the clinical relevance of cognitive impairment in patients with liver disease.

## Conflict of Interest

The authors of this manuscript declare no conflicts of interest.

## Supporting information


**Data S1**. NIH Toolbox cognition battery.
