# Supplementary material for: Impaired cognition predicts the risk of hospitalization and death in cirrhosis
Source: Ann Clin Transl Neurol. 2019 Oct 20;6(11):2282–90. doi: 10.1002/acn3.50924 (PMC6856598; doi:10.1002/acn3.50924)
Supplement: Supplementary file 1 — Data S1. NIH Toolbox cognition battery. [file ACN3-6-2282-s001.docx]

**The NIH Toolbox Cognition Battery**

The Pattern Comparison Processing Speed test (PCPS) assesses choice reaction time by asking patients to determine whether two visual patterns are identical by touching a “Yes” or “No” button on the iPad screen. The raw score is the total correct responses out of 130. The List Sorting Working Memory test (LSWM) consists of several trials wherein complementary visual and audio stimuli (animals and foods; e.g., screen shows image of banana and audio says “BANANA” aloud), presented one at a time, and participants are asked to repeat the items in order of size from smallest to largest. An additional stimulus is added after each list is answered correctly, and the test is discontinued when two lists of the same length are answered incorrectly. The raw score is the total number of lists repeated correctly. Flanker Inhibitory Control and Attention test (FICA) examines patients’ ability to inhibit visual attention to distractor arrows (flankers) and focus on a central stimulus arrow. Across multiple trials, the arrows change directions, varying between trials where the stimulus and flanker arrows face the same direction (congruent) and where they face opposite directions (incongruent). The raw score is calculated from the response accuracy and response time over 40 trials. In Dimensional Change Card Sort test (DCCS), participants match a target visual stimulus to 1 of 2 choice stimuli by color or shape. Raw scores are calculated from the participant’s reaction time.
